# New Separation Material Obtained from Waste Rapeseed Cake for Copper(II) and Zinc(II) Removal from the Industrial Wastewater

**DOI:** 10.3390/ma14102566

**Published:** 2021-05-14

**Authors:** Krzysztof Mazurek, Sebastian Drużyński, Urszula Kiełkowska, Edward Szłyk

**Affiliations:** Faculty of Chemistry, Nicolaus Copernicus University in Toruń, 7 Gagarina Street, 87-100 Toruń, Poland; sebdru@umk.pl (S.D.); ulak@chem.umk.pl (U.K.); eszlyk@chem.umk.pl (E.S.)

**Keywords:** waste rapeseed cake, sulphuric(VI) acid, waste acids, wastewater, adsorption, removal, copper, zinc, arsenic, biochar

## Abstract

Rapeseed cake biochar was produced by pyrolysis at 973.15 K for 2 h, in anoxic conditions. Porous structure, specific surface area and die composition of waste rapeseed cake were studied. The specific surface area of rapeseed cake biochar was 166.99 m^2^·g^−1^, which exceeded most other biochars reported, which made it an attractive material during wastewater treatment. The SEM study of the material demonstrated a large number of pores formed on the cell wall, with a pore volume *V_p_* = 0.08 cm^3^·g^−1^. The results indicate lower aromaticity and increased polarity of the tested material. The observed H/C ratio of 0.29 is similar for activated carbons. Furthermore, sorption properties of the obtained carbon material in relation to copper(II), zinc(II) and arsenic(III) ions were also studied. Moreover, the impact of parameters such as: sorption time, temperature, adsorbate concentration, sorbent mass and solution pH on the efficiency of the adsorption process of the studied cations was also examined. Sorption studies revealed that the sorbent can be successfully used for the separation of Cu(II) and Zn(II) from technological wastewaters. Rapeseed cake biochar exhibits superior Cu(II) adsorption capacity (52.2 mg·g^−1^) with a short equilibrium time (6 h). The experimental data collected show a high selectivity of the obtained carbon material relative to copper(II) and zinc(II) ions in the presence of arsenic(III) ions.

## 1. Introduction

Protection of the natural environment as well as natural resources has become the greatest challenge faced by humanity in the first decades of the 21st century. The expansion of industry, technology and science, as well as the growth of the human population around the world, have a huge impact on the condition of the natural environment and commonly observed climate changes [1,2,3].

The development of industrial production is strongly associated with the rapid shrinkage of natural resources. Moreover, industrial manufacturing causes the emission and production of increased amounts of solid, liquid and gaseous waste.

One of the most important elements of sustainable development is the maximisation of the process efficiency and reduction in losses of natural resources. The easiest way to follow the aforementioned above idea is the recycling of waste. It has to be underlined that such wastes could become a valuable source of numerous resources in industry. Recycling in line with the 3R principle (reduce, reuse, recycle) is a key activity that allows us to reduce the amount of industrial waste and decrease the exploitation of natural resources in a significant degree [4].

The activities mentioned above have become the key objectives of all EU policy areas, as demonstrated by Directive 2008/98/EC. The current EU regulations favour technological and apparatus solutions enabling a reduction in the generated waste amount and regulating the system of waste management [5].

Sulphuric(VI) acid is currently produced in three types of industrial installations: metallurgical, sulphuric and catalytic wet. The production of sulphuric(VI) acid in any technology leads to the generation of industrial waste. The volume and variety of the residues are determined by the type of installation applied. The largest amounts of wastes are undoubtedly produced in metallurgical installations. Waste acidic scrubber liquids, produced during the process, are recognised as liquid wastes. They are formed in the gas purification stage during the sulphuric(VI) acid production process performed directly from the metallurgical area [6,7,8,9].

The quantity and chemical composition of generated waste acidic scrubber liquids depend on the following factors: type of technology applied during non-ferrous metal ores processing, the composition of concentrates, the efficiency of dry process gas dedusting and technology of wet cleaning with process gas. However, it should be underlined that sulphuric(VI) acid production, based on the usage of process gases from the non-ferrous metal ores metallurgical unit, produces from 0.2 to 1.4 m^3^ of waste acid per 1 Mg of manufactured monohydrate. The concentration of the impurities also varies ranging from a few to several dozen percent (Table 1) [6,7,8,9].

Nowadays, the precipitation of poorly soluble sulphides in the dynamic reactors technology is applied in order to clean off waste acids during sulphuric(VI) acid production [6,7,8,9].

The greatest challenge in the waste acid utilisation process is the presence and composition of arsenic compounds. In a typical procedure, arsenic is separated by the precipitation of the less soluble sulphides or arsenates. However, valuable components such as copper and zinc are co-precipitated during the standard procedure. Arsenic has a negative impact on the pyrometallurgical process of copper and zinc and the natural environment. It poses a severe problem in the technological process of SO_2_ utilisation as sulfuric(VI) acid due to the poisoning of the vanadium catalyst and a reduction in the quality and commercial value of the produced acid. The arsenic-containing wastes should not be reused in the metallurgical plant [6,7,8,9].

Research into alternative methods of acidic scrubber liquids purification, enabling the separation of valuable components from arsenic compounds, is important from the point of view of technology and environmental protection. Several methods (reverse osmosis, ultrafiltration, electrodialysis and ion exchange) are described in the literature with the potential to be applied for acidic waste treatment [6,7,8,9]. There is also a necessity to search for new adsorption materials enabling one to obtain higher separation efficiency of individual waste components of acidic scrubber liquids. The efficiency and quality of the separated phase has a decisive impact on the recovery rate of copper(II) and zinc(II) ions, which can be recycled to the pyrometallurgical process.

In recent years, adsorbents derived from agricultural and industrial wastes received an extensive attention due to their wide availability, low cost and physicochemical properties. Biochars are regarded as promising metals adsorbents due to their abundance of polar functional groups. The aim of this work was to investigate the methodology of a new carbon material preparation procedure from rapeseed cake. Moreover, the scope of the work was physicochemical characterisation of the obtained material. The new material will be applied for the adsorption and separation of copper(II), zinc(II) and arsenic(III) ions from acidic scrubber liquids in order to recover Cu(II) and Zn(II) from wastewater. To the best of our knowledge, there are no reports concerning biochar production from waste rapeseed cake and its sorption properties. The studies of Ozcimen and Karaosmanoglu concern biochar production at low temperature (773 K); however, they do not involve sorption characteristics studies [10].

## 2. Experimental Part

### 2.1. Reagents and Apparatus

Reagents of analytical purity grade purity: copper(II) sulphate(VI) pentahydrate, ≥99.0 wt.%; zinc(II) sulphate(VI) heptahydrate, ≥99.0 wt.%; sulphuric(VI) acid, ≥95.0 wt.%; sodium hydroxide, ≥99.0 wt.% (Avantor Performance Materials, Gliwice, Poland); arsenic trioxide, ≥99.0 wt.% (UCB S.A, Anderlecht, Belgium); ammonium persulphate, ≥98 wt.% (Merck, Darmstadt, Germany) were applied in the study. Waste rapeseed cake was kindly provided by a local manufacturer (Prem-Vit Sp. J., Inowrocław, Poland).

To characterise the solid phase, the following apparatuses were used: FEI’s scanning electron microscope Quanta 3D FEG (SEM, Fei Company, Cambridge, UK), TA Instruments’ SDT 2960 (TGA-DTA, TA Instruments, New Castle, UK), Micromeritic’s sorptomat ASAP 2010 (BET, Micromeritics Instrument Corporation, Norcross, GA, USA), Elementar’s Vario MACRO CHN Cube (CHN, Elementar Analysensysteme GmbH, Hanau, Germany), and Malvern Panalytical’s MasterSizer 3000 (particle size, Malvern Panalytical, Malvern, UK).

The adsorption experiments were conducted in a thermostatic bath constant with the Polystat CC1 thermorelay (±0.1 K). The set temperature was controlled with the use of a mercury thermometer with an accuracy of ±0.1 K.

PANalytical’s MiniPal 4 compact energy dispersive X-ray spectrometer (Malvern Panalytical, Malvern, UK) was employed to determine the concentrations of Cu, Zn and As in solution and for the qualitative analysis of the solid phase.

Elmetron’s multifunctional CX-742 device (Elmetron, Zabrze, Poland) equipped with Ionode’s Ion44C combination electrode was employed for pH measurements.

### 2.2. Preparation and Characterisation of Biochar Adsorbent

**Figure 1:**
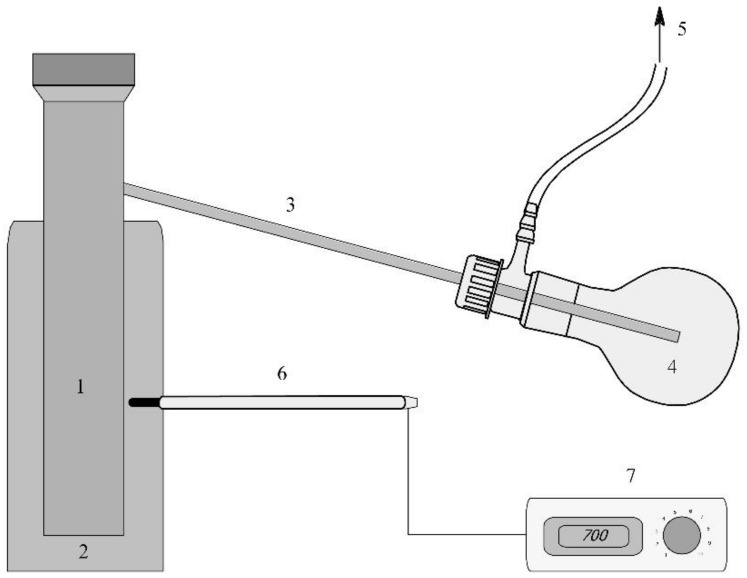
The apparatus used for pyrolysis of the rapeseed cake (1. pyrolysis reactor, 2. furnace. 3. air cooler, 4. condenser, 5. waste gases, 6. thermocouple, 7. temperature controller).

Raw rapeseed cake was treated with 40% solution of ammonium peroxodisulphate at a weight ratio of 1:1 prior to pyrolysis. Subsequently, the prepared mixture was dried for 72 h at ambient temperature. The mixture prepared in this way was subjected to pyrolysis conducted in no air system at 673.15, 773.15 and 973.15 K in the apparatus set (Figure 1). The sample was heated up to the set temperature at a heating rate of 10 K·min^−1^. The process was continued for about 1 h at the set temperature and obtained char was left to cool. The obtained biochar was ground in a planetary ball mill and subjected to purification in a Soxhlet apparatus, where it was washed with acetone and n-heptane. Subsequently, it was dried at 378 K and used for the adsorption study.

### 2.3. Adsorption Procedure

The adsorption study was conducted with the use of standard solutions of copper(II) sulphate(VI) and zinc(II) sulphate(VI) with concentrations of 50–300 mg·dm^−3^. For the prepared standard solutions, optimal adsorption parameters were determined: pH 0–5 range adjusting using concentrated sulphuric(VI) acid, sorbent mass 1.0–3.3 g·dm^−3^, adsorption temperature 298.15–323.15 K, contact time of 5–480 min.

The appropriate amount of biochar adsorbent and adsorbate at a certain concentration were used for adsorption study. Samples in 100 cm^3^ Erlenmeyer flasks with a rubber stopper were placed in a thermostat. The adsorption study of the samples was performed at a constant temperature, in the range 298.15–323.15 K, for 5 to 480 min upon stirring. After the process completion, the contents of the flasks were filtered and 20 cm^3^ of a filtrate was transferred quantitatively into a 25 cm^3^ volumetric flask and fill up to the mark with distilled water. The prepared samples were analysed quantitatively for Cu(II) and Zn(II) ions.

The efficiency of the adsorption process in relation to the tested cations was calculated from Equation (1).

(1) $$A\;[\%]\;=\;\frac{C^{o}\;-\;C^{e}}{C^{o}}\;\times \;100\%$$

where *c^o^* and *c^e^* are the initial and equilibrium concentrations [mg·dm^−3^] of the analysed ion, respectively.

Moreover, the kinetics of the adsorption process was studied. For this purpose, adsorption was conducted with the use of 0.1 g of adsorbent and 30 cm^3^ of copper(II) or zinc(II) solutions at various concentrations (100–300 mg·dm^−3^ and 50–200 mg·dm^−3^, respectively, in 5–480 min intervals, at a temperature of 298.15–323.15 K. The equilibrium capacity of the adsorbent (q_e_) [mg·g^−1^] was calculated according to Equation (2).

(2) $$q_{e}\;=\;\frac{\left(C^{o}\;-\;C^{e}\right)\;\times \;V}{m}$$

where *V* is the volume of the solution, and *m* is the mass of the adsorbent.

The selectivity of the tested biochar in relation to Cu(II), Zn(II) and As(III) ions was determined. The adsorption experiment was conducted with the use of 0.1 g adsorbent and 30 cm^3^ of copper(II), zinc(II) and arsenic(III) standard solutions with concentrations of 50, 100 and 200 mg·dm^−3^, respectively. The experiment was performed for 240 min at 313.15 K. The adsorption efficiency was calculated using Equation (1).

### 2.4. Analytical Methods

The concentrations of all ions in the solutions were measured with the use of the EDXRF method. All measurements were made in triplicate and averaged. This method might be applied for the ion concentration range between 1 ppm and 100%.

The solid phase was characterised physicochemically by the EDXRF, SEM, TGA–DTA, and BET techniques.

## 3. Results and Discussion

### 3.1. Adsorbent Characterisation

The results of CHN elementary analysis (Table 2) indicate that the carbon content in the obtained sorbent did not exceed 65% along with a relatively large amount of nitrogen (above 5%). The obtained biochar contains a higher carbon concentration than reported by Ozcimen and Karaosmanoglu [10], most probably due to the differences in the waste preparation method and pyrolysis conditions. The degree of carbonisation expressed by the H/C molar ratio, according to the data in Table 2, indicates clearly that a pyrolysis temperature increase resulted in the H/C molar ratio systematically decreasing. The observed H/C ratio, 0.29 (973.15 K), is lower than the value obtained for the biochar studied by Ozcimen and Karaosmanoglu, 0.47 [10], and similar to that of the activated carbon, 0.26, obtained by Chen et al. [11]. These low H/C ratios suggest that the discussed biochar is strongly carbonised and does not contain organic residues for rape plant, such as cellulose. On the other hand, the relatively high content of nitrogen (5.6%) in biochar may also have a positive effect on the adsorption process. Nitrogen can form highly polar functional groups on the surface of the biochar, resulting in greater hydrophilicity of the surface.

**Figure 2:**
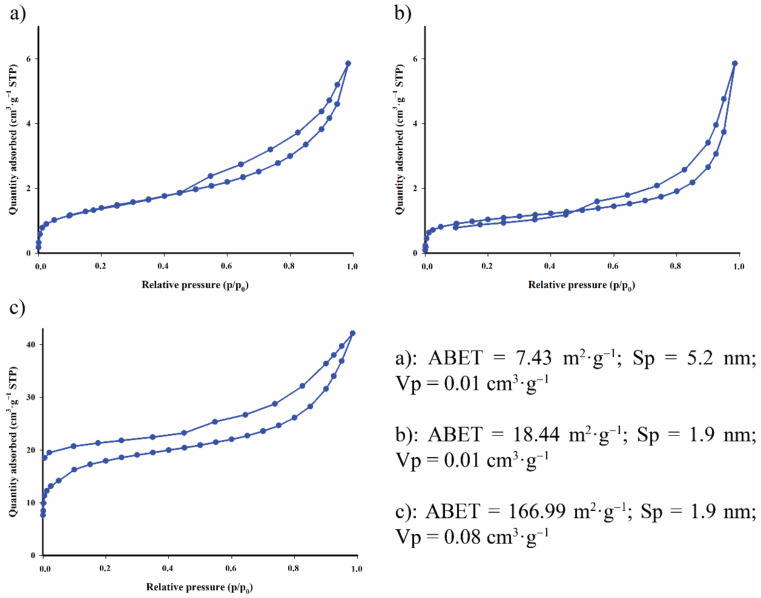
N_2_ adsorption/desorption isotherm of carbon material obtained at 673.15 K (**a**), 773.15 K (**b**) and 973.15 K (**c**).

The nitrogen adsorption–desorption isotherms are shown in Figure 2. The observed curves indicate that the surface area of the studied biochar increases systematically with increasing pyrolysis temperature. The literature data (Wang et al. [12]) indicate that when the preparation temperature is equal to or above 873 K, the specific surface area of the obtained biochar is larger than that of most other materials. This is most probably due to the gradual evaporation and breakdown of volatile organic components as the pyrolysis temperature was increased from 673 to 973 K, resulting in the formation of a microporous structure.

The biochar obtained at 973.15 K characterises the mean pore diameter (*S_p_*), calculated according to the Barrett–Joyner–Halenda (BJH) algorithm, as being equal to 1.9 nm (Figure 2c). The carbon material was also characterised by surface area of *A_BET_* = 166.99 m^2^·g^−1^ and pore volume *V_p_* = 0.08 cm^3^·g^−1^. Nitrogen adsorption/desorption studies revealed that the obtained isotherm is of type IV, according to International Union of Pure and Applied Chemistry (IUPAC) classification, indicating the existence of mesopores.

Wang et al. demonstrated that the specific surface area of biochar, produced from other precursors (peanut shell, sludge, bamboo reed, etc.) at 573–973 K, was in the range of 3.75–54.05 m^2^·g^−1^ [13]. In this experiment, the specific surface area of biochar prepared from waste rapeseed cake was much larger. The latter confirms the correct choice of pyrolysis conditions and biochar activation method.

Based on the obtained results, the biochar at the highest temperature of pyrolysis (973.15 K) revealed the most promising physicochemical properties of metal ion adsorption, and hence the material was chosen for further studies.

**Figure 3:**
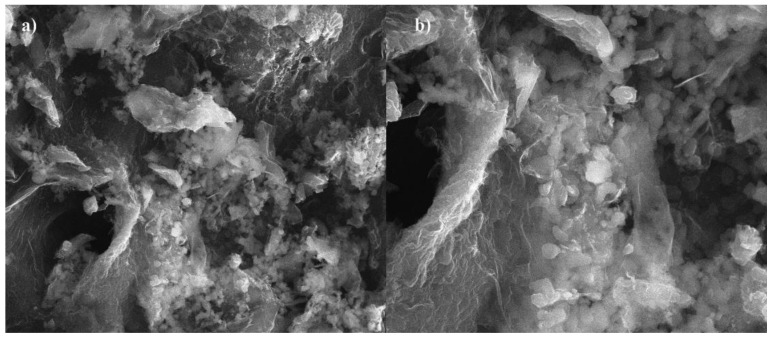
SEM images of biochar in the scale of (**a**) 10 μm and (**b**) 4 μm.

SEM images of the sorption material are presented at Figure 3. The prepared carbon sorbent is composed of irregularly shaped particles of varying grain sizes that tend to agglomerate. The external surface of the obtained biochar also has deep slits, which could be formed upon the removal of volatile organic matter during pyrolysis.

**Figure 4:**
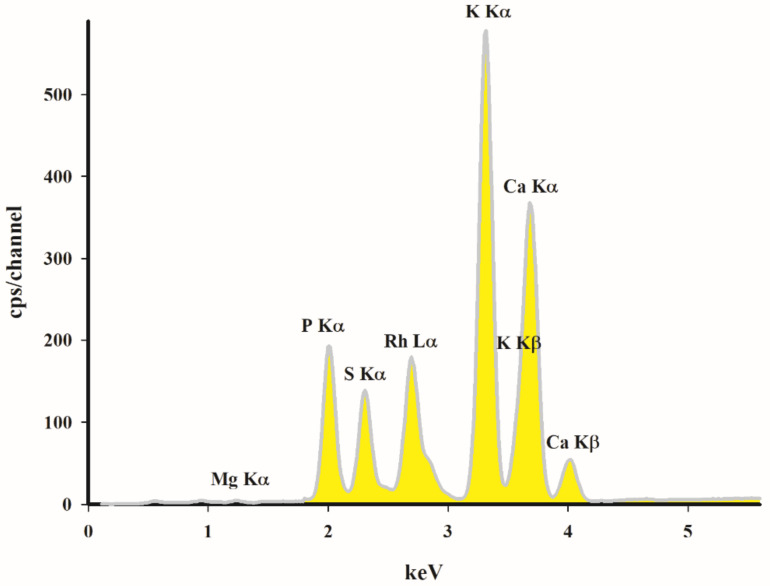
EDXRF spectrum of studied carbon biochar sorbent.

The EDXRF analysis (Figure 4) revealed that the studied porous material possesses significant amounts of potassium, calcium, phosphorus and magnesium. The presence of these metals in biochar is beneficial for the metal adsorption process. They can exchange or precipitate with the studied metals and reduce their availability. The reported adsorption kinetic results indicate that the adsorption of heavy metals by the cation exchange mechanism in the biochar occurs at the early stage of the reaction. Park et al. and Lu et al. reported that the effects of cation exchange on the adsorption of heavy metals for rice straw and sludge biochar were equal to 62.3% (Cu^2+^) and 51.8% (Pb^2+^), respectively [14,15].

**Figure 5:**
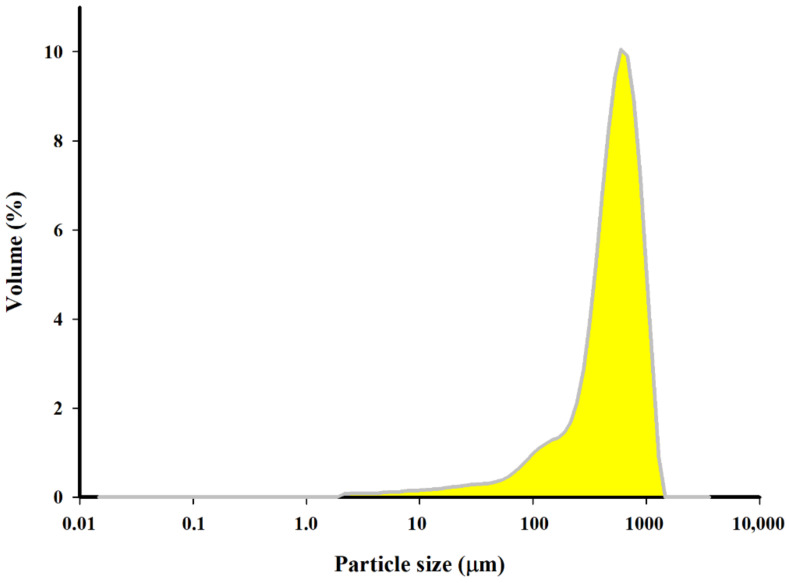
Particle size distribution of the biochar.

Granulometric analysis of the samples suspended in methanol was performed with a MasterSizer 3000 laser apparatus. The obtained biochar revealed the dominant fraction with a size of 119–844 μm and an average specific surface of 45.2 m^2^·kg^−1^ (Figure 5).

**Figure 6:**
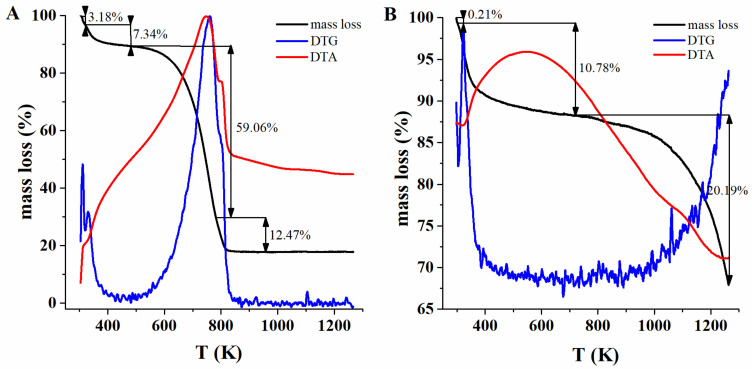
Thermogravimetric analysis: (**A**) air atmosphere; (**B**) nitrogen atmosphere.

TGA-DTA analysis was carried out in air and in a nitrogen atmosphere (100 cm^3^·min^−1^) and with a heating rate of 5 K·min^−1^. The results are listed in Table 3, while TGA-DTA curves are plotted in Figure 6. The TG curve (Figure 6A) of the sample exhibits four stages.

In the first endotherm, 304.6–321.3 K moisture is released. The second endotherm (321.3–468.5 K) can be related to the degradation of an unstable oxygen species (i.e., carboxyl, hydroxyl or lactone groups,) along with further loss of water. In the third exoenergetic stage (468.9–792.0 K), 59.06% of mass loss is noted due to the nitrogen-containing groups decomposition and destruction of the sample carbon material’s microstructure. The fourth stage (792.0–1273.2 K) can be assigned to the decomposition of the most thermally stable sulphur groups. The TG curve recorded under a nitrogen atmosphere (Figure 6B) is a three-stage process. In the first stage, the sample loses a small amount of adsorption water (299.2–311.5 K). The second stage (311.5–754.0 K) (Figure 6B) can be caused by the sample decomposition after treatment with acetone and n-heptane, which unblocks the pores and causes the detachment of oxygen and nitrogen functional groups. In the third stage (onset 754.0 K), sulphur groups most probably decompose on the surface of biochar.

The obtained results of the TGA-DTA tests clearly indicate that oxygen, nitrogen and sulphur groups are present on the surface of the obtained biochar. This is closely related to the method of raw material modification prior to the pyrolysis process with the use of ammonium peroxodisulphate. The presence of various functional groups on the surface of the sorbent significantly improves the sorption capacity and significantly extends the potential application time of the material.

### 3.2. Adsorption Process

**Figure 7:**
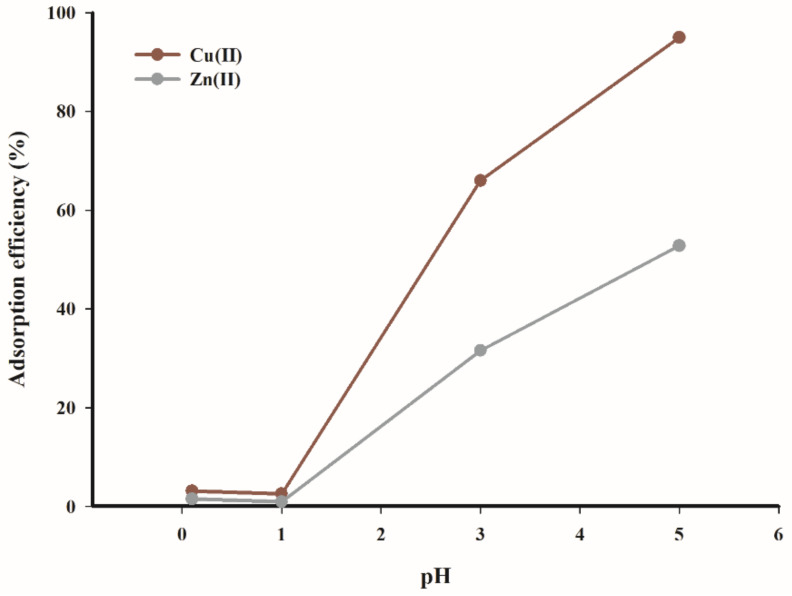
Effect of pH on the adsorption efficiency (T = 298.15 K, t = 240 min, c = 100 mg·dm^−3^, mass of adsorbent = 3.33 g·dm^−3^).

The effect of pH on the adsorption rate of copper(II) and zinc(II) ions was tested and obtained results are presented in Figure 7. The data in Figure 7 clearly demonstrate that the adsorption of copper(II) and zinc(II) ions was not favourable at low pH (0–3). The calculated difference in adsorption efficiency in the pH range of 0.1–5 was equal to 90%, while the highest adsorption rate (95%) was obtained for a solution of pH 5.

In the case of zinc(II) ions, the significant rise in the adsorption efficiency was observed at pH = 5. At same time, the highest process performance, below 53%, was obtained.

The observed dependencies are consistent with the results reported by Hawari and Mulligan, as well as Rahman and Islam for the adsorption of copper cations on various natural sorbents, which determine the optimal pH range as 4–5.5 [16,17]. Similar results were obtained by Gao et al. during an investigation of the kinetics and mechanism of copper(II) ion adsorption on an activated carbon obtained from pinewood sawdust [18]. The optimum pH was in the range 4–6 for copper(II) ions; in a strongly acidic medium, the reaction proceeds with less efficiency due to the protonation of functional groups on the sorbent surface. At a low pH, both metal cations were adsorbed to a lesser extent on the charged surface of biochar due to electrostatic repulsions. An increase in pH caused a charge change on the sorbent surface, generating more negatively charged groups. Furthermore, the number of competing hydronium ions decreased in an environment with an increase in pH value.

The analysis of data presented in Figure 7 indicates that the tested biochar adsorbent exhibits higher affinity for copper(II) ions than for zinc(II) ions.

**Figure 8:**
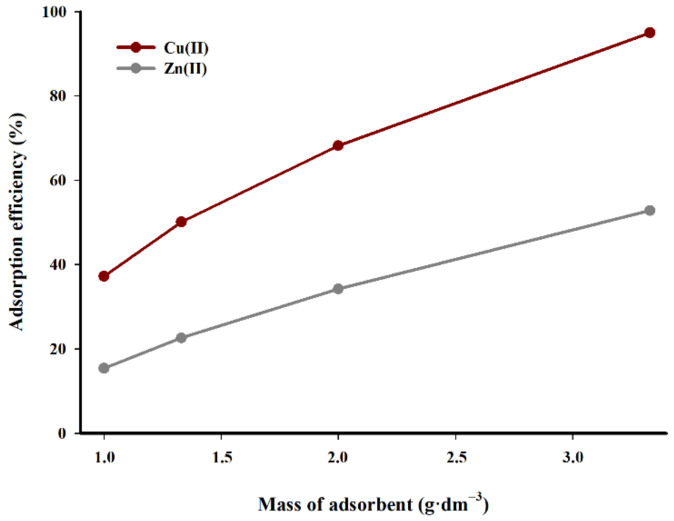
Effect of the adsorbent mass (g·dm^−3^) on the adsorption efficiency (T = 298.15 K, t = 240 min, c = 100 mg·dm^−3^, pH = 5).

The studies of the effect of sorbent concentration (g·dm^−3^) on the efficiency of the adsorption of copper(II) and zinc(II) are presented in Figure 8. The research performed by different authors has demonstrated that the ratio of adsorbent to the solution volume has a considerable impact on the efficiency of the adsorption process [19,20,21,22]. The efficiency value increases with the increase in adsorbent mass.

Analogous relations can be observed in Figure 8. It is evident that a higher concentration of a sorbent in the suspension improves the adsorption efficiency of copper(II) cations. The increase in sorbent concentration in the suspension from 1.0 to 3.33 g·dm^−3^ results in a performance increase of almost 60%, while in the case of zinc, the adsorption efficiency is less pronounced. The efficiency of the process increases linearly with the rise in the mass of the adsorbent used in relation to the volume of solution. The difference in adsorption efficiency between the limit tested sorbent concentrations in suspension is over 37%. It can be assumed that when optimising the sorption efficiency, the concentration of sorbent in the suspension should oscillate within 3 g·dm^−3^.

**Figure 9:**
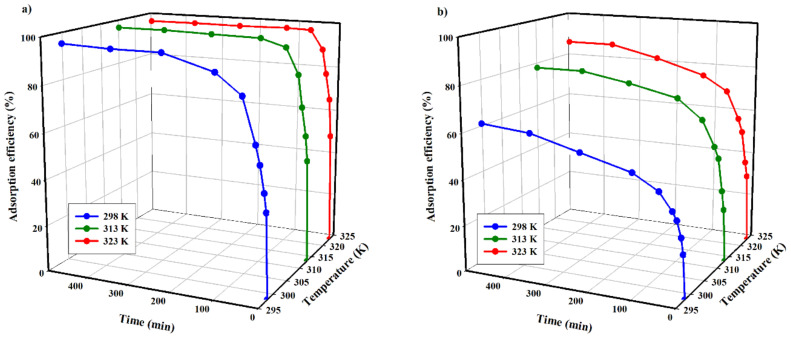
Adsorption efficiency as a function of time and temperature: (**a**) copper(II); (**b**) zinc(II); (c = 100 mg·dm^−3^, mass of adsorbent = 3.33 g·dm^−3^, pH = 5).

The efficiency of copper(II) and zinc(II) adsorption on the tested carbon adsorbent is presented in Figure 9.

The analysis of data shown in Figure 9 indicates that efficiency values are affected by the temperature increase for both tested cations. The lowest efficiency was obtained for the experiments performed at the lowest temperature (298.15 K), while the maximum efficiency for copper(II) and zinc(II) ions was 96 and 63%, respectively. At 323.15 K, the values of the process efficiency were the highest for both cations, at 98 and 87%, respectively.

At the same time, it is clear that the temperature of the process impacts the adsorption of zinc(II) ions to a larger extent than copper(II) ions. In the case of Zn^2+^, increasing the process temperature by 25 K results in an increase in efficiency of about 21%. An analogous increase in process temperature, in the case of Cu^2+^, results in a 2% larger process efficiency.

Furthermore, it is evident that the adsorption process temperature results in a shortened time necessary to reach thermodynamic equilibrium in the system. In the case of Zn(II), adsorption at 298.15 K, during an 8 h experiment, appeared to be too low to reach an equilibrium state. Increasing the process temperature by 15 K results in achieving equilibrium after approximately 6 h, while a further temperature increase by 10 K does not reduce the equilibrium time.

Such thermodynamic dependence of the copper(II) and zinc(II) adsorption process on temperature can be related to its endothermic nature. This may be attributed to an increased penetration of the studied cations inside micropores at higher temperatures or the creation of new active sites. The endothermic adsorptions may be due to a stronger interaction between pre-adsorbed water and the adsorbent than the interaction between copper(II) and zinc(II) ions and the adsorbent.

The temperature increase may impact the kinetic energy of both cations, which in turn may lead to an increase in their mobility and promote spontaneous adsorption. On the other hand, the temperature increase causes the solution viscosity to decrease, which results in a rise in ionic diffusion rate and faster adsorption to the surface of the tested biochar. The endothermic adsorption of the studied cations on biochar appears to be uncommon behaviour. However, several authors have reported endothermic adsorption of heavy metal cations on different types of adsorbents of natural origin [23,24].

Considering the technological nature of the research, the recommended temperature of adsorption seems to be ca. 313.15 K. It should be noted that the temperature of acidic scrubber liquid waste fluctuates within this temperature.

**Figure 10:**
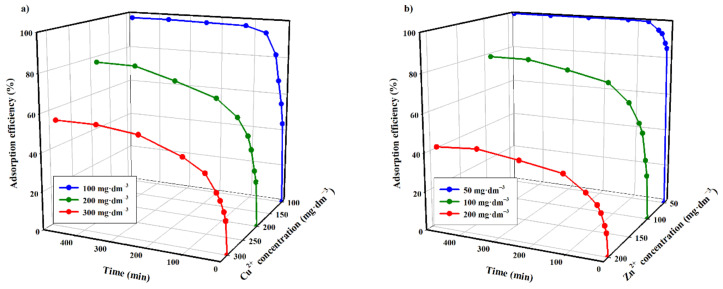
Adsorption efficiency as a function of time and initial concentration (**a**) copper(II); (**b**) zinc(II) (T = 313.15 K, mass of adsorbent = 3.33 g·dm^−3^, pH = 5).

The next stage of research involved the determination of the optimal time for the adsorption process. Studies were conducted for standard solutions with initial concentrations of copper(II) and zinc(II) ions of 100, 200 and 300 mg·dm^−3^, as well as 50, 100 and 200 mg·dm^−3^, respectively (Figure 10).

The adsorption progress indicates that a high degree of removal of the tested ions was achieved after two hours of the adsorbent contact with the adsorbate (Figure 10). The efficiency of the adsorption process for copper(II) ions after this contact time was 97, 65 and 45%, respectively, for increasing initial concentrations of Cu^2+^ in solutions. The analysis of curves in Figure 10 indicates that the increase in the initial concentration of Cu^2+^ ions from 100 to 300 mg·dm^−3^ reduces the adsorption efficiency by approximately 52%.

Analogous studies of zinc(II) adsorption indicate that the same time of contact with the adsorbate resulted in the adsorption efficiency of 100, 71 and 38%, respectively, for increasing initial concentrations of Zn^2+.^ Here, there is also a substantial impact of the concentration of the researched cation in the solution on the efficiency of the adsorption in the studied carbon adsorbent. The increase in Zn^2+^ concentration from 50 to 200 mg·dm^−3^ results in an adsorption efficiency decrease of ca. 62%, in relation to starting zinc(II) concentration (50 mg·dm^−3^).

The equilibrium point for both cations, depending on the initial concentration, was reached relatively slowly. In the case of a copper(II) cation solution of 100 mg·dm^−3^, the thermodynamic equilibrium was found after ca. two hours of the adsorption process, while in the case of solutions of concentration of 200 and 300 mg·dm^−3^, the equilibrium time was reached after 6 h. For the initial zinc(II) ion concentration (50 mg·dm^−3^), the equilibrium point was reached after 60 min, whereas at 200 mg·dm^−3^ the time was extended to ca. 6 h.

The data show that the adsorption equilibrium of metal ions reported by others is much slower for carbon sorbents when compared to inorganic, hybrid or ion exchange resin adsorbents [20,25,26,27].

**Figure 11:**
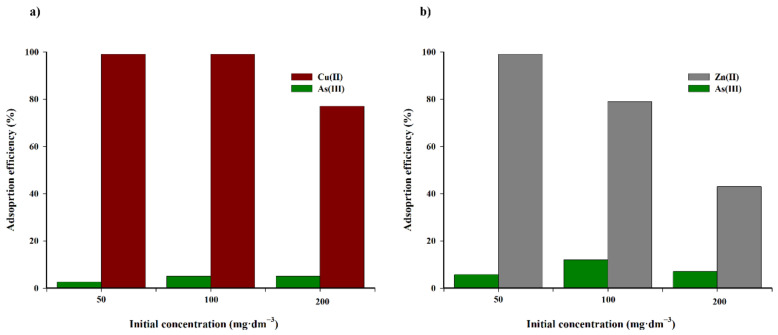
Effect of initial concentration on the adsorption efficiency of copper(II) (**a**), zinc(II) (**b**) and arsenic(III) ions (T = 313.15 K, t = 240 min, mass of adsorbent = 3.33 g·dm^−3^, pH = 5).

To conclude this part of the research, the selectivity of the biochar sorbent is effective for a mixture of copper(II) and zinc(II) in the presence of arsenic(III) ions in the range of 50–200 mg·dm^−3^, which is presented in Figure 11.

Arsenic(III) ions are adsorbed to a small extent from the solution on the tested biochar. Furthermore, the presence of arsenic(III) in the solution only has a slight impact on the increase in the adsorption rate of copper(II) and zinc(II) cations.

**Figure 12:**
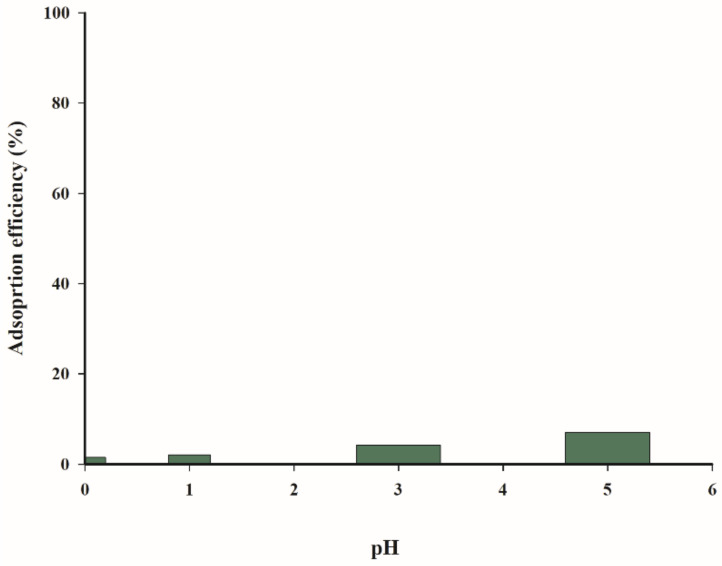
Effect of pH on the adsorption efficiency of arsenic(III) (T = 298.15 K, t = 240 min, c = 100 mg·dm^−3^, mass of adsorbent = 3.33 g·dm^−3^).

The solution acidity does not increase the adsorption of arsenic(III) ions in the studied pH range (1–5), because of a constant low level of only a few to several percent (Figure 12).

### 3.3. Adsorption Kinetics Study

On the basis of the experimental data, kinetic models were calculated in order to precisely design the adsorption process. For that purpose, the equation of the pseudo first-order by the Lagergren kinetic model and Ho’s equation of pseudo-second order kinetic model were applied.

The linear model of pseudo-first order kinetics is defined according to Equation (3):

(3) $$\log\left(q_{e}\;-\;q_{t}\right)\;=\;\log q_{e}\;-\;k_{1}\;\times \;t$$

where *q_e_* and *q_t_* define the amounts of metal ions adsorbed in a point of equilibrium and at time *t* (min), and *k*_1_ (1·min^−1^) is the speed constant for the pseudo-first order model.

The calculated kinetics parameters for the adsorption of studied metal ions onto the biochar, at initial concentration of 100 and 200 mg·dm^−3^, are listed in Table 4.

The linear correlation coefficients indicate that the first-order kinetic model of pseudo-first order adsorption provides a satisfactory description of the adsorption of copper(II) and zinc(II) cations on the tested sorbent. However, the adsorption capacity results based on this kinetic model q_e,cal_ are substantially different from the values obtained experimentally in all cases.

The linear model of the pseudo-second order equation by Ho is:

(4) $$\frac{t}{q_{t}}\;=\;\frac{1}{k_{2}\;\times \;q_{e}^{2}}\;+\;\frac{1}{q_{e}}\;\times \;t$$

where *q_e_* and *q_t_* determine the amounts of metal cations adsorbed at a point of equilibrium and at time *t* (min), and *k*_2_ (g·mg^−1^·min^−1^) is the speed constant for the pseudo-second order model, respectively.

**Figure 13:**
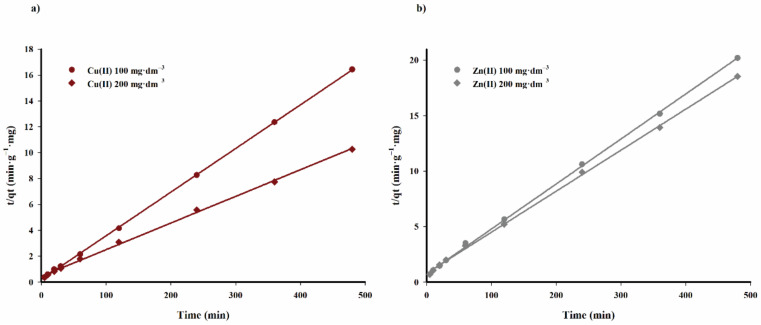
Pseudo-second-order kinetic fit for adsorption of copper(II) (**a**) and zinc(II) (**b**) cations of carbon adsorbent.

The values of the constant rate of adsorption process *k*_2_ and calculated adsorption capacity q_e,calc._ for specific concentrations of lithium and cobalt(II) ions in solution are listed in Table 5, calculated on the basis of straight lines shown in Figure 13.

The data listed in Table 5 indicate that the pseudo-second order kinetic model accurately describes the kinetics of the adsorption process of copper(II) and zinc(II) cations on the tested biochar sorbent. The adsorption capacity values calculated on the basis of the discussed model are highly correlated with the experimental data.

The adsorption rate constant *k*_2_ decreases with an increasing concentration of copper(II) ions in the solution. However, the effect of zinc(II) cation concentration in the solution on this parameter is minimal.

The parameters listed in Table 4 and Table 5 clearly show that the pseudo-second order kinetic model reflects the kinetics of the investigated process better and can be sufficient to predict the adsorption of copper(II) and zinc(II) cations on the tested biochar sorbent. The presented results are consistent with the works of Demiral and Gungor as well as Adebisi et al. [28,29]. The process of adsorption of copper(II) and zinc(II) cations on carbon sorbents presented in these works is also best described by pseudo-second order kinetics.

To assess the adsorption capacity and the sorption mechanism of the biochar, the Langmuir and Freundlich isotherm models were applied.

The Langmuir isotherm equation is given by the equation:

(5) $$\frac{c_{e}}{q_{e}}\;=\;\frac{1}{K\;\times \;q_{m}}\;+\;\frac{c_{e}}{q_{m}}$$

where *q_e_* (mg·g^−1^) is the real adsorption at sorption equilibrium, *c_e_* (mg·dm^−3^) is the concentration of cation in the solution at equilibrium point, *q_m_* (mg·g^−1^) is the maximum adsorption on the sorbent surface, and *K* (dm^3^·mg^−1^) is a constant associated with the adsorption energy.

Freundlich isotherm adsorption on heterogeneous (energetically heterogeneous) surfaces and on microporous adsorbents is given by equation:

(6) $$q_{e}\;=\;K_{F}\;\times \;c_{e}^{1/n}$$

where *q_e_* (mg·g^−1^) is the real adsorption at sorption equilibrium, *c_e_* (mg·dm^−3^) is the concentration of cation at equilibrium, *K_F_* (mg·g^−1^) is a constant, expressing maximum adsorption on the sorbent surface, and 1/*n* is a characteristic constant related to the intensity of the adsorption process. The linear form of this equation is most often used for calculations:

(7) $$logq_{e}\;=\;\log K_{F}\;+\;1/n\;\times \;\log c_{e}$$

**Figure 14:**
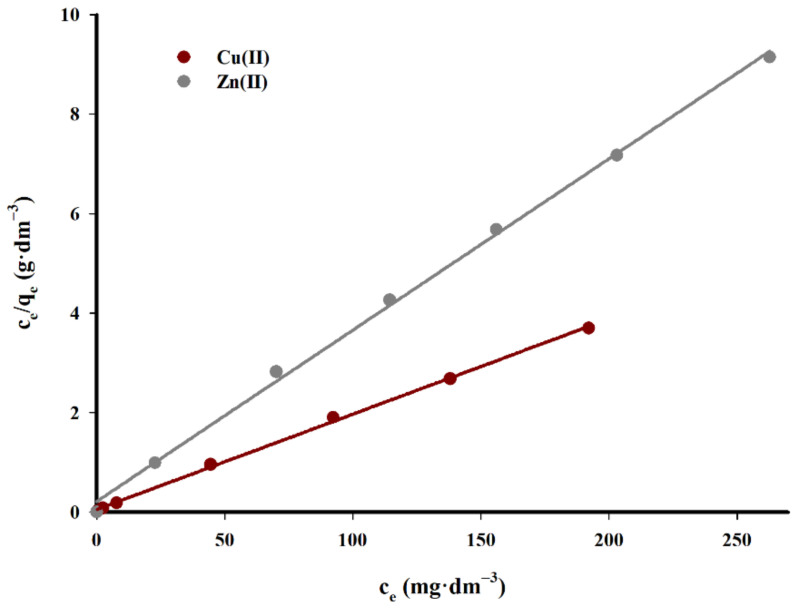
Langmuir isotherm fit for adsorption of copper(II) and zinc(II) ions onto studied sorbent.

**Figure 15:**
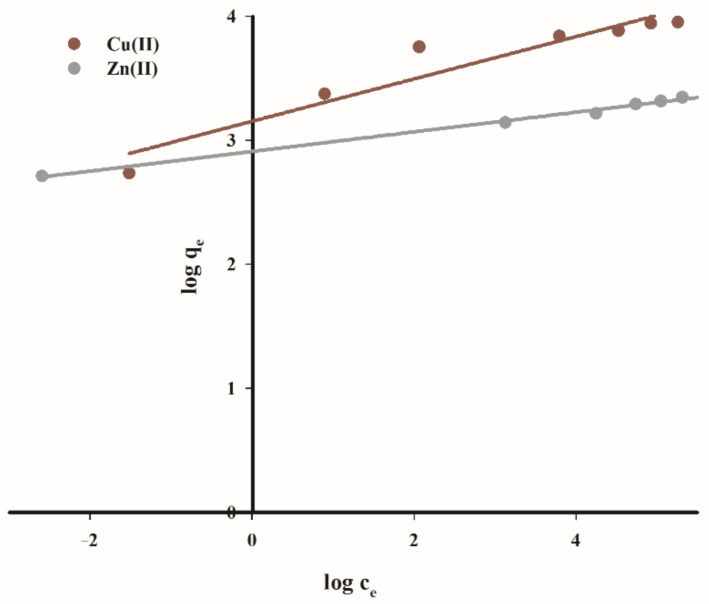
Freundlich isotherm fit for adsorption of copper(II) and zinc(II) ions onto studied adsorbent.

Figure 14 and Figure 15 show a graph of studied isotherms for the sorption of copper(II) and zinc(II) cations on the tested carbon sorbent, while in Table 6 the calculated parameters of constants and values R^2^ for both considered isotherms are listed.

As demonstrated by the presented data, the adsorption of copper(II) on the tested carbon sorbent is best reflected by the Langmuir isotherm. It has been proved that the sorbent used has a varied surface structure, and during the adsorption process reactions occur mainly in the monolayer with no additional interactions that could give rise to the effects of multilayer adsorption.

The maximum sorption capacity calculated for the studied carbon biochar sorbent for copper(II) ions is 52.2 mg·g^−1^.

In the case of zinc(II) cations, a very good fit of straight lines was obtained for both tested models with a slight predominance of Langmuir isotherms. The maximum sorption capacity in for zinc(II) ions is was 29.0 mg·g^−1^.

The comparison of the selected maximum adsorption capacities for various types of carbon sorbents are listed in Table 7 and Table 8. A comparison of the data in Table 7 and Table 8 indicates that the sorbent obtained from waste rapeseed cake by pyrolysis reveals a relatively good value of sorption capacity in relation to copper(II) and zinc(II) ions. The advantage of the studied biochar is also high selectivity in relation to the ion mixture of Cu^2+^/As^3+^ and Zn^2+^/As^3+^. The high level of separation permits the recovery of these metals, which is undoubtedly beneficial from an ecological and economic point of view.

The arsenic remaining in waste acidic scrubber liquids after sorption should also be removed. The best method appears to be the precipitation of arsenic in the form of sparingly soluble compounds. Battaglia-Brunet et al. suggested the precipitation of arsenic in the form of arsenic sulphide with high efficiency [38]. According to Grzesiak, arsenic can also be precipitated in the form of calcium arsenate, chloro-arsenate of lead and iron arsenate [9].

**Figure 16:**
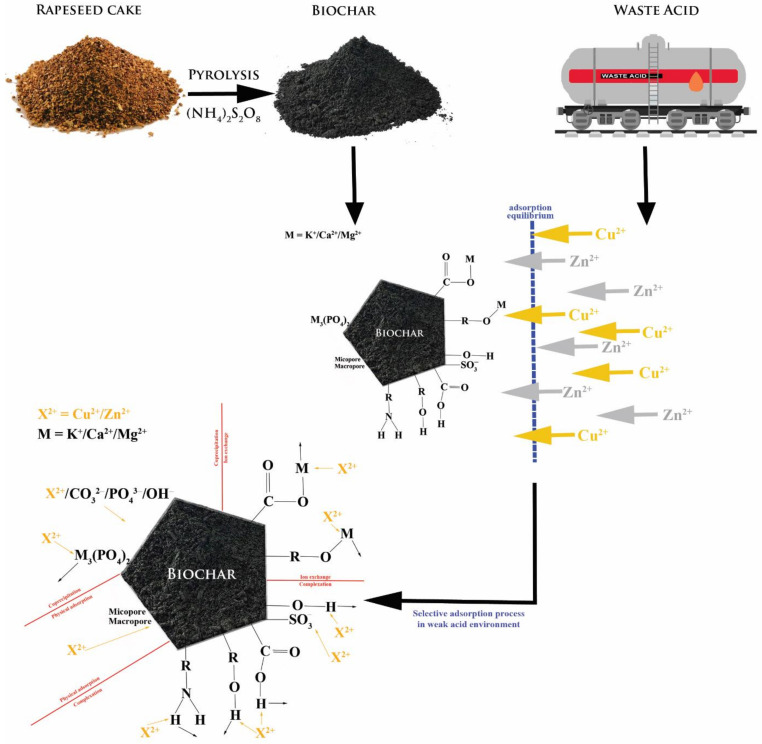
Proposed mechanism of adsorbent/adsorbate interactions.

Based on the experimental data, the mechanism of adsorbent/adsorbate interactions was proposed (Figure 16). It should be emphasised that these interactions are mostly affected by the pH of the reaction. On the other hand, the interactions seem to be caused by the electrostatic and chemical nature.

## 4. Conclusions

The study presents the properties of carbon sorbent obtained by pyrolysis of waste rapeseed cake. The proposed method of managing the wastes of the agricultural industry enables us to obtain sorption material with relatively high sorption parameters. The obtained carbon material has a specific surface (A_BET_) of 166.99 m^2^·g^−1^ and pore volume (*V_p_*) equal to 0.08 cm^3^·g^−1^.

The obtained material is characterised by a relatively large affinity to copper(II) and zinc(II) ions and very low affinity towards arsenic(III) ions, which has been confirmed by the experimental results. The adsorptive performance of the proposed adsorbent mainly depends on the time of contact between the adsorbate and adsorbent, the adsorption temperature, the cation initial concentration, the weight of the sorbent used in relation to the volume and the pH of the solution.

The Langmuir isotherm model reflects the sorption of copper(II) cations on the tested carbon sorbent better, ensuring a higher linear correlation coefficient (R^2^ = 0.999), compared to Freundlich isotherm (R^2^ = 0.911), and the maximum adsorption capacity of 52.2 mg·g^−1^. In the case of zinc(II), the Langmuir isotherm model also provides a slightly better linear correlation coefficient (R^2^ = 0.998), compared to Freundlich isotherm (R^2^ = 0.995), and the maximum adsorption capacity *q_m_* = 29.0 mg·g^−1^. The research on adsorption kinetics reveals that the process occurs on the tested carbon sorbent in accordance with the pseudo-second order kinetic equation.

## Figures and Tables

**Table 1:** Content of copper, zinc, iron and arsenic ions in waste acidic scrubber liquids.

Element	Copper Metallurgy	Zinc Metallurgy
	Content (ppm)	
Cu	1–200	-
Zn	-	1–300
As	2–1000	0–500
Fe	10–40	0–0.5

**Table 2:** Carbon, hydrogen, and nitrogen elemental analysis results of biochar as a function of pyrolysis temperature.

Element	Content (wt.%)				
	673.15 K		773.15 K		973.15 K
C	61.80		62.34		63.30
H	2.54		2.27		1.51
N	7.12		6.18		5.63

**Table 3:** TGA-DTA analysis results of biochar sample.

Atmosphere Air			
Step	Mass Loss (%)	Energy Effect	Temperature Range (K)
1	3.18	endo	304.6–321.3
2	7.34	endo	321.3–468.9
3	59.06	exo	468.9–792.0
4	12.47	exo	792.0–1273.2
**Atmosphere N_2_**			
1	0.21	endo	299.2–311.5
2	10.78	exo	311.5–754.0
3	20.19	endo	754.0–1273.2

**Table 4:** Pseudo-first-order kinetic parameters obtained by linear method at different copper(II) and zinc(II) cation concentrations.

Parameter		Cations Concentration (mg·dm^−3^)			
		Copper(II)		Zinc(II)	
Symbol	Unit	100	200	100	200
q_e,exp._	mg·g^−1^	29.2	46.8	23.8	26.0
k_1_	1·min^−1^	0.017	0.013	0.013	0.011
R^2^	-	0.93	0.98	0.98	0.98
q_e,calc._	mg·g^−1^	8.9	34.2	14.7	16.5

**Table 5:** Pseudo-second-order kinetic parameters obtained by linear method at different copper(II) and zinc(II) cations concentration.

Parameter		Cations Concentration (mg·dm^−3^)			
		Copper(II)		Zinc(II)	
Symbol	Unit	100	200	100	200
q_e,exp._	mg·g^−1^	29.2	46.8	23.8	26.0
*k*_2_	g·mg^−1^·min^−1^	0.005	0.001	0.002	0.002
R^2^	-	0.99	0.99	0.99	0.99
q_e,calc._	mg·g^−1^	29.6	48.6	24.7	27.1

**Table 6:** Freundlich and Langmuir isotherms parameters for adsorption of copper(II) and zinc(II) cations onto studied sorbent.

Isotherm						
Ion	Freundlich			Langmuir		
	R^2^	*K_F_* (mg·g^−1^)	*n*	R^2^	*q_m_* (mg·g^−1^)	*K* (dm^3^·mg^−1^)
Cu^2+^	0.911	1417.4	5.858	0.999	52.196	0.360
Zn^2+^	0.995	809.1	12.642	0.998	29.043	0.160

**Table 7:** Adsorption capacities of different adsorbents towards removal of copper(II) ions from aqua-solutions.

Adsorbent	*q_m_* (mg·g^−1^)	Reference
Biochar	52.20	This study
Hydrochar	48.22	Semercioz et al. (2017) [30]
Manure biochar	44.50	Batool et al. (2017) [19]
Manure biochar	44.50	Idrees et al. (2018) [31]
Activated carbon	43.47	Demiral and Gungor (2016) [28]
Activated carbon fibers	35.24	Qiao et al. (2020) [32]
Pomegranate peel biosorbent	30.12	Ben-Ali et al. (2017) [20]
Graphene oxide	26.75	Zhang et al. (2016) [33]
Biochar	15.70	Cibati et al. (2017) [22]
Rapeseed waste	15.43	Tofan et al. (2011) [34]
Hardwood biochar	4.39	Jiang et al. (2016) [35]

**Table 8:** Adsorption capacities of different adsorbents towards removal of zinc(II) ions from aqua-solutions.

Adsorbent	*q_m_* (mg·g^−1^)	Reference
Rice straw biochar	38.6	Park et al. (2017) [14]
Biochar	35.75	Sanyang et al. (2014) [36]
Biochar	29.04	This study
Rapeseed waste	13.86	Paduraru et al. (2015) [37]
Corn straw biochar	11.0	Chen et al. (2011) [21]
Biochar	10.4	Cibati et al. (2017) [22]
Hardwood biochar	2.31	Jiang et al.(2016) [35]

## Data Availability

Data sharing is not applicable to this article.

## References

[1] Fan Y., Wu S., Lu Y., Zhao Y. (2019). Study on the Effect of the Environmental Protection Industry and Investment for the National Economy: An Input-Output Perspective. J. Clean. Prod..

[2] Kavanagh J. (1994). Environmental Protection and Waste Minimization: A Case Study. J. Clean. Prod..

[3] Fugiel A., Burchart-Korol D., Czaplicka-Kolarz K., Smoliński A. (2017). Environmental Impact and Damage Categories Caused by Air Pollution Emissions from Mining and Quarrying Sectors of European Countries. J. Clean. Prod..

[4] Das S., Lee S.-H., Kumar P., Kim K.-H., Bhattacharya S.S. (2019). Solid Waste Management: Scope and the Challenge of Sustainability. J. Clean. Prod..

[5] Lazarevic D., Buclet N., Brandt N. (2012). The Application of Life Cycle Thinking in the Context of European Waste Policy. J. Clean. Prod..

[6] Grzesiak P., Grobela M., Motała R. (2013). Ecological Aspects and Development Strategies of Sulfuric Acid Industry. Przem. Chem..

[7] Grzesiak P., Grobela M., Motała R., Łukaszyk J., Schroeder G., Cichy B. (2011). Strategy for Dealing with Waste Materials in the Production of Sulfuric Acid. Przem. Chem..

[8] Grzesiak P. (2010). Trend of Sulfuric Acid Production in Metallurgical Installations. Chemik.

[9] Grzesiak P. (2004). Development of Sulfuric Acid Production in Poland.

[10] Özçimen D., Karaosmanoğlu F. (2004). Production and Characterization of Bio-Oil and Biochar from Rapeseed Cake. Renew. Energy.

[11] Chen B., Zhou D., Zhu L. (2008). Transitional Adsorption and Partition of Nonpolar and Polar Aromatic Contaminants by Biochars of Pine Needles with Different Pyrolytic Temperatures. Environ. Sci. Technol..

[12] Wang Z., Shen D., Shen F., Wu C., Gu S. (2017). Kinetics, Equilibrium and Thermodynamics Studies on Biosorption of Rhodamine B from Aqueous Solution by Earthworm Manure Derived Biochar. Int. Biodeterior. Biodegrad..

[13] Wang P., Zhan S., Yu H., Xue X., Hong N. (2010). The Effects of Temperature and Catalysts on the Pyrolysis of Industrial Wastes (Herb Residue). Bioresour. Technol..

[14] Park J.-H., Wang J.J., Kim S.-H., Cho J.-S., Kang S.-W., Delaune R.D., Han K.-J., Seo D.-C. (2017). Recycling of Rice Straw through Pyrolysis and its Adsorption Behaviors for Cu and Zn Ions in Aqueous Solution. Colloids Surf. A Physicochem. Eng. Asp..

[15] Lu H., Zhang W., Yang Y., Huang X., Wang S., Qiu R. (2012). Relative Distribution of Pb2+ Sorption Mechanisms by Sludge-Derived Biochar. Water Res..

[16] Hawari A.H., Mulligan C.N. (2006). Biosorption of Lead(II), Cadmium(II), Copper(II) and Nickel(II) by Anaerobic Granular Biomass. Bioresour. Technol..

[17] Rahman M.S., Islam M.R. (2009). Effects of pH on Isotherms Modeling for Cu(II) Ions Adsorption Using Maple Wood Sawdust. Chem. Eng. J..

[18] Gao X., Wu L., Xu Q., Tian W., Li Z., Kobayashi N. (2018). Adsorption Kinetics and Mechanisms of Copper Ions on Activated Carbons Derived from Pinewood Sawdust by Fast H3PO4 Activation. Environ. Sci. Pollut. Res..

[19] Batool S., Idrees M., Hussain Q., Kong J. (2017). Adsorption of Copper (II) by using Derived-Farmyard and Poultry Manure Biochars: Efficiency and Mechanism. Chem. Phys. Lett..

[20] Ben-Ali S., Jaouali I., Souissi-Najar S., Ouederni A. (2017). Characterization and Adsorption Capacity of Raw Pomegranate Peel Biosorbent for Copper Removal. J. Clean. Prod..

[21] Chen X., Chen G., Chen L., Chen Y., Lehmann J., McBride M.B., Hay A.G. (2011). Adsorption of Copper and Zinc by Biochars Produced from Pyrolysis of Hardwood and Corn Straw in Aqueous Solution. Bioresour. Technol..

[22] Cibati A., Foereid B., Bissessur A., Hapca S. (2017). Assessment of Miscanthus × Giganteus Derived Biochar as Copper and Zinc adsorbent: Study of the Effect of Pyrolysis Temperature, pH and Hydrogen Peroxide Modification. J. Clean. Prod..

[23] Liu Z., Zhang F.-S. (2009). Removal of Lead from Water using Biochars Prepared from Hydrothermal Liquefaction of Biomass. J. Hazard. Mater..

[24] Tan X., Liu Y., Zeng G., Wang X., Hu X., Gu Y., Yang Z. (2015). Application of Biochar for the Removal of Pollutants from Aqueous Solutions. Chemosphere.

[25] Li Q., Fu L., Wang Z., Li A., Shuang C., Gao C. (2017). Synthesis and Characterization of a Novel Magnetic Cation Exchange Resin and its Application for Efficient Removal of Cu 2+ and Ni 2+ from Aqueous Solutions. J. Clean. Prod..

[26] Maleki A., Hajizadeh Z., Sharifi V., Emdadi Z. (2019). A Green, Porous and Eco-Friendly Magnetic Geopolymer Adsorbent for Heavy Metals Removal from Aqueous Solutions. J. Clean. Prod..

[27] Bouhamed F., Elouear Z., Bouzid J., Ouddane B. (2016). Multi-Component Adsorption of Copper, Nickel and Zinc from Aqueous Solutions onto Activated Carbon Prepared from Date Stones. Environ. Sci. Pollut. Res..

[28] Demiral H., Güngör C. (2016). Adsorption of Copper(II) from Aqueous Solutions on Activated Carbon Prepared from Grape Bagasse. J. Clean. Prod..

[29] Adebisi G.A., Chowdhury Z.Z., Alaba P.A. (2017). Equilibrium, Kinetic, and Thermodynamic Studies of Lead Ion and Zinc Ion Adsorption from Aqueous Solution onto Activated Carbon Prepared from Palm Oil Mill Effluent. J. Clean. Prod..

[30] Semerciöz A.S., Göğüş F., Çelekli A., Bozkurt H. (2017). Development of Carbonaceous Material from Grapefruit Peel with Microwave Implemented-Low Temperature Hydrothermal Carbonization Technique for the Adsorption of Cu (II). J. Clean. Prod..

[31] Idrees M., Batool S., Kalsoom T., Yasmeen S., Kalsoom A., Raina S., Zhuang Q., Kong J. (2018). Animal Manure-Derived Biochars Produced via Fast Pyrolysis for the Removal of Divalent Copper from Aqueous Media. J. Environ. Manag..

[32] Qiao K., Yu J., Zhu B., Chi C., Di C., Cheng Y., Shang M., Li C. (2020). Oxygen-Rich Activated Carbon Fibers with Exceptional Cu(II) Adsorptivity and Recycling Performance. Ind. Eng. Chem. Res..

[33] Zhang K., Li H., Xu X., Yu H. (2016). Facile and Efficient Synthesis of Nitrogen-Functionalized Graphene Oxide as a Copper Adsorbent and Its Application. Ind. Eng. Chem. Res..

[34] Tofan L., Paduraru C., Volf I., Toma O. (2011). Waste of Rapeseed from Biodiesel Production as a Potential Biosorbent for Heavy Metal Ions. BioResorces.

[35] Jiang S., Huang L., Nguyen T.A., Ok Y.S., Rudolph V., Yang H., Zhang D. (2016). Copper and Zinc Adsorption by Softwood and Hardwood Biochars under Elevated Sulphate-Induced Salinity and Acidic pH Conditions. Chemosphere.

[36] Sanyang L., Ghani W.A.W.A.K., Idris A., Mansor A. (2014). Zinc Removal from Wastewater Using Hydrogel Modified Biochar. Appl. Mech. Mater..

[37] Paduraru C., Tofan L., Teodosiu C., Bunia I., Tudorachi N., Toma O. (2015). Biosorption of Zinc(II) on Rapeseed Waste: Equilibrium Studies and Thermogravimetric Investigations. Process. Saf. Environ. Prot..

[38] Battaglia-Brunet F., Crouzet C., Burnol A., Coulon S., Morin D., Joulian C. (2012). Precipitation of Arsenic Sulphide from Acidic Water in a Fixed-Film Bioreactor. Water Res..

